# Health, life and rights: a discourse analysis of a hybrid abortion regime in Tanzania

**DOI:** 10.1186/s12939-019-1039-6

**Published:** 2019-09-27

**Authors:** Richard Sambaiga, Haldis Haukanes, Karen Marie Moland, Astrid Blystad

**Affiliations:** 1 Department of Sociology and Anthropology, University of Dar es Salaam, Dar es Salaam, Tanzania; 2 Department of Health Promotion and Development, University of Bergen, Bergen, Norway; 3 Global Health Anthropology Research Group, Centre for International Health, Department of Global Public Health and Primary Care, University of Bergen, Bergen, Norway; 4 Centre for Intervention Science in Maternal and Child Health (CISMAC), University of Bergen, Bergen, Norway

**Keywords:** Abortion law, Abortion policy, Discourses on abortion, Access to safe abortion services, Tanzania, East- Africa

## Abstract

**Background:**

Unsafe abortion continues to be a major hazard for maternal health in Sub-Saharan Africa, where abortion remains highly controversial and access to safe abortion services is unequally distributed. Although national abortion laws are central in indicating women’s potential for accessing safe abortion services, the character of an abortion law may alone say little about national discursive abortion landscapes and access scenarios. The article calls for the study and problematization of the relationship between legal abortion frameworks on the one hand, and discourses surrounding abortion on the other, in an attempt to move closer to an understanding of the complexity of factors that influence knowledge about and access to safer abortion services. With the restrictive abortion law in Tanzania as a starting point, the paper explores the ways in which the major global abortion discourses manifest themselves in the country and indicate potential implications of a hybrid abortion regime.

**Methods:**

The study combined a review of major legal and policy documents on abortion, a review of publications on abortion in Tanzanian newspapers between 2000 and 2015 (300 articles), and 23 semi-structured qualitative interviews with representatives from central institutions and organizations engaged in policy- or practical work related to reproductive health.

**Results:**

Tanzania’s abortion law is highly restrictive, but the discursive abortion landscape is diverse and is made manifest through legal- and policy documents and legal- and policy related disputes. The discourses were characterized by diverse frames of reference based in religion, public health and in human rights-based values, and as such reflect the major global discourses. Fairclough’s concepts *interdiscursivity* and *recontextualization* were drawn upon to develop an understanding of how the concepts health, rights and life emerge across the discourses, but are employed in contrasting lines of argumentation in struggles for hegemony and legitimacy.

**Discussion and conclusions:**

The paper demonstrates that a hybrid discursive regime relating to abortion characterizes the legally restrictive abortion context of Tanzania. We argue that such a complex discursive landscape, which cuts across the restrictive - liberal divide, generates an environment that seems to open avenues for enhanced access to abortion related knowledge and services.

## Introduction

Induced abortion continues to be a highly controversial topic in large parts of the world, and debates surrounding abortion are often rife with controversies. Globally competing normative discourses on abortion have been well documented in the literature on sexual and reproductive health [1–5], and in the aftermath of the reinstatement of the Mexico City Policy or ‘global gag rule’ by the US President in 2017 the topic has again been located centrally in global political discourse. The challenge of unsafe abortion is of enormous scale, and is held to be one of the most neglected sexual and reproductive health problems in the world today. With 19–20 million estimated cases of unsafe abortion annually, it caters for some 68,000 annual deaths and a substantial proportion of the continued high maternal death- and morbidity rates globally [6].

Unsafe abortion has been tightly linked to restrictive abortion laws and lack of access to contraception and safe abortion services. United Nations [7] reported in 2014 that the average unsafe abortion rate was more than four times higher in countries with restrictive abortion policies than in countries with liberal abortion policies. Countries with restrictive policies moreover had a maternal mortality ratio that was three times higher than in countries with liberal abortion policies. The dynamics between national abortion laws and women’s actual knowledge about and access to abortion services is nonetheless not straight forward, and at times emerges as both ambiguous and paradoxical [1, 4, 8, 9].

This article presents findings from a study that explored the discursive abortion landscape within the legally restrictive abortion context of Tanzania with the aim to generate knowledge on the relationship between the law, the prevailing abortion debates in the country and their potential implications for access to abortion related knowledge and services. The literature on abortion from Tanzania has focused on incidence, abortion seeking practices, and service delivery [10–13]. Although there is a serious lack of quality data, the available figures indicate that about 16% of maternal deaths in Tanzania are due to complications from abortion, and unsafe abortion is estimated to be the second leading cause of maternal deaths in Tanzania [14–16]. A related regional figure indicates that 18% of maternal deaths are linked to unsafe abortion in Eastern Africa [17, 18]. The challenge of unsafe abortion thus emerges as substantial in the region, not least among the younger population. There are a few published studies related to post abortion care [17–19], a service that also the government of Tanzania has committed to. Comprehensive Post Abortion Care (PAC) are as such introduced also in countries with restrictive abortion laws. Reliable data on abortion incidence is not available, but the table below (Table 1) sums up major maternal health indicators from Tanzania, and indicates the continued high maternal mortality ratio, fertility rate and unmet need for family planning on the one hand, and the still low contraceptive rates on the other.

**Table 1:** Maternal health indicators.

Maternal mortality ratio*	Fertility rate**	Contraceptive prevalence girls 15–19*	Contraceptive prevalence 15–49*	Unmet need for family planning*
398	5.0	14.9	34.4	25.3

*WHO 2019 Global health observatory country views [20]

**The World Bank 2017 https://data.worldbank.org/indicator/sp.dyn.tfrt.in [21]

Several studies have noted that there are diverse and contrasting abortion debates in Tanzania, and that global abortion discourses are reflected at national level [22–25]. No study has to date explored these discourses in depth in a Tanzanian context. As public abortion discourses shape the contexts within which women and girls live their (reproductive) lives, increasing knowledge on the manner in which global abortion discourses are played out within a restrictive legal context is of substantial importance. The present study sought to scrutinize the proponents and content of the prevailing abortion debates in Tanzania, and explored how the discourses are produced, reproduced and how they contrast and intersect. Two central questions were raised: how are claims for or against abortion framed in different abortion discourses in Tanzania? What spheres of potential competition and intersection emerge in the process of negotiating hegemony and legitimacy of the respective discourse and with what implications? These questions remain at the margin of the growing literature on abortion, but make up a vital sphere of knowledge to enhance the understanding of the contexts that frame and shape women and girls’ knowledge about access to abortion.

In our approach to the study of the discursive landscape on abortion in Tanzania we draw upon the work of Norman Fairclough, who since the late 1980s has published extensively on Critical Discourse Analysis. In this paper we do not carry out a full-fledged critical discourse analysis, but the study is informed by Fairclough’s key concepts *discourse*, *interdiscursivity* and *recontextualization* [26, 27]. Discourse is a complex concept and there are many overlapping and partly conflicting definitions that are formulated from various theoretical and disciplinary standpoints. According to Fairclough [27:164] “Discourses are semiotic ways of construing aspects of the world (physical, social or mental) which can generally be identified with different positions or perspectives of different groups or social actors”, and he emphasizes that discourses can be ‘operationalized or ‘put into practice’. Interdiscursivity points to the implicit or explicit relations that one discourse – or an aspect of a discourse - has to another. Fairclough argues that ‘interdiscursivity’ calls for an understanding of the multiplicity of frames of reference of a particular discourse, emphasizing the grasping of its context. He moreover demonstrates how discourses which originate in some particular social field or institution may be recontexualizerd in others [27:165]. Interdiscursivity is as such closely related to and dependent upon the concept of recontextualisation*.* Discursive elements that originate in one discursive regime may be ‘re-contextualized’ in others as a means to ‘colonize’ one regime by another, or as ‘appropriation’ of external elements. This may take place as an incorporation of discursive elements into strategies pursued by particular groups of social agents in struggles for hegemony and legitimacy [26, 27]. In the present article we draw upon the concepts discourse, interdiscursivity and recontextualization in efforts to enhance the understanding of how different abortion related debates and positions are played out and manifest themselves in a Tanzanian context. In the paper, we moreover employ the concept ‘hybrid regime’, a concept often applied in political science to refer to political regimes that combine democratic and autocratic traits [28, 29]. In this particular context hybrid regime points to the co-existence of diverging discourses that govern the knowledge about and practices related to abortion.

## Methods

The paper draws on material collected in a qualitative study conducted within the framework of a three-year regional study (2016–19) focusing on competing discourses which affect girls’ and women’s rights to fertility control and safe abortion in Zambia, Ethiopia and Tanzania. For this paper, data was collected from 2016 to 2018 in Tanzania. Attempts at capturing the content of abortion discourses is clearly a demanding task, and can hardly be done without biases and limitations. Given the complexity and sensitivity of the topic of induced abortion, methods triangulation seemed important. We included the systematic studies of; 1) the abortion law and abortion-related policy; 2) media coverage on the abortion issue and 3) qualitative interviews with individuals centrally located within the field of abortion.

In Tanzania, newspapers were found to be a good source to track abortion discourses as they bring together issues reported in other media channels, especially television, radio and social media, and they can be systematically searched. Almost all radio and television stations have a programme that highlights key issues covered by the mainstream newspapers. The search for material from the newspapers entailed developing a checklist of abortion and fertility-control related topics such as anti-abortion initiatives by pro-life organizations, religious leaders or government officials, abortion-related cases reported to the police or the court system and initiatives by NGOs to discuss or promote access to fertility control or abortion services. A systematic search was made for these topics in the main daily Tanzanian newspapers; Mwananchi, Mtanzania, Habari Leo, Uhuru, Nipashe, Daily News, The Citizen and The Guardian. Some 300 articles on abortion were published between 2000 and 2015.

Semi-structured interviews were conducted with a total of 23 key informants. The informants represented different institutions, organizations and positions within the abortion debates. The varied sources provided us with an opportunity to approach the topic from diverse angles. The material was collected in Dar es Salaam region which was considered an important study area as most of the relevant institutions e.g. ministries, UN agencies, international and local NGOs, and religious organizations shaping the discourses on fertility control and abortion in Tanzania are located in this major urban hub. The institutions and organizations contacted were selected on the basis of their involvement in reproductive health related activity, including abortion related policy, interventions or advocacy for or against abortion. Informants from the Ministry of Health, Community Development, Gender, Elderly and Children (MIN 1); the Ministry of Constitutional and Legal Affairs (MIN 2); organizations working on sexual and reproductive health such as UN organizations (UN); international NGOs (INGO), local NGOs (NGO), religious pro-life organizations, and Christian and Muslim leaders (RO) were included. Six (6) health providers (HP) from public and private health facilities in Dar es Salaam were moreover included in the study. The inclusion of the health workers was done to gain a sense of health providers’ knowledge and opinions about the national abortion law and how they relate to the prevailing abortion discourses in the country. Ethnographic material from research on abortion among women and girls in Dar es Salaam, collected as part of the broader study of which this sub-study is part, is in the publication process (Solheim, forthcoming).

The authors are academics/researchers in the social sciences and employed at national universities in Tanzania and Norway. The team has extensive experience from research on a variety of sexual and reproductive health issues from Tanzania. In our engagement with informants we chose not to clarify our own positions on the sensitive abortion issue in efforts to facilitate a positive and open atmosphere that encouraged engagement and reflection. The informants were interested in the study topic, and eagerly engaged in the description and discussion of their institutions’ abortion related positions, activities and impact. The interviews were conducted in English and Swahili, and were, with some exceptions (4 in number), audio recorded, and subsequently transcribed. Rapid handwritten notes were made during the interviews that were not audio recorded.

The analysis process started during the review of legal and policy documents, the newspaper coverage and the first interviews, while a more rigorous analysis phase took place post fieldwork. Nvivo 11 software was used as a tool to store, review and organize the material from the diverse sources. The process involved numerous rounds of reading and re-reading of the full data set and the identification of the content related to the main abortion discourses through processes of coding and categorization. The key notions health, life and rights emerged across and framed all the major discourses.

Ethics: The informants were informed about the focus of the research both in writing and orally. The research ethical principles of voluntary consent, rights of withdrawal, confidentiality and anonymity were rigorously adhered to. All informants provided oral consent to participate in the research. For anonymity reasons we do not mention the names of the organizations approached. The research project had clearance from the Norwegian Centre for Research Data (57,089/3/00SIRH), and from the University of Dar es Salaam (CoSS- SO18011).

### Study findings

Findings from the research demonstrate that the three relatively distinct and dominant discourses found on the global arena, namely the anti-abortion discourse, and the two pro-safe abortion discourses - one based in public health and the other in human rights - were encountered as active and alive also in the Tanzanian context. The three discourses broadly position themselves either in line with or against the conservative Tanzanian abortion law. Below we start out by presenting the legal framework for abortion in Tanzania, pieces of related Constitutional texts as well as relevant global treaties that Tanzania is a signatory to. Thereafter we demonstrate how diverse discourses emerge in policy documents, media postings and in statements by representatives from organizations and institutions revealing how they are played out, including in policy disputes. In the process we make attempts to demonstrate how the discourses are produced, reproduced, justified and legitimized in opposition or in alliance with one another.

### Tanzanian abortion-related law and policy

The Tanzanian abortion law is inscribed in the Penal Code and implied in the country’s Constitution. Like in most African countries, the origin of the restrictive abortion law can be traced back to the colonial legacy, notably to the English legal codes [30–32]. The Tanzanian penal code criminalizes illegal abortion as an “offense against morality” [33] (chapter XV). It provides grounds for punishing the person who unlawfully facilitates an abortion, the woman who procures her own abortion, and the one who supplies drugs or instruments with the intent to procure an abortion. Section 219 of the Penal Code frames abortion as “child destruction” - as an offense connected to murder, and those convicted as liable to life imprisonment. Abortion is legally permitted by the law in Tanzania in defense of the health and life of a pregnant woman, and states that in such circumstances a person is not criminally responsible for performing abortion in good faith and with reasonable care and skills (Section 230 of the Penal Code of Tanzania, Cap.16 R.E, 2002).

The table below (Table 2) sums up the content of the Tanzanian abortion law.

**Table 2:** The abortion law in Tanzania.

Legal status of abortion	Illegal, highly restrictive
Grounds for legal abortion	
1.To save the life of the woman	1. Yes
2.To preserve physical/mental health	2. No
3. Rape or incest	3. No
4. Foetal impairment	4. No
5. Economic/social reasons incl.	5. No
Who decides that abortion can be legally performed?	One health care worker can decide without consulting others, profession not specified
Where can abortion be performed?	Health facility, level not specified
Procedural guidelines for safe abortion services	No
Criminal punishment for violating restrictions	Abortionist: Imprisonment up to 14 years Woman: Imprisonment up to 7 years Others involved: Imprisonment up to 3 years

Table reference: The Penal Code of Tanzania Cap.16, section 230 [33].

Newspaper excerpts suggest that the legal framework detailed in the abortion law is from time to time referred to by politicians and bureaucrats. A typically emerging example is a statement of the Prime Minister who in 2016 issued a strong warning to medical practitioners alleged to be facilitating abortion in public health facilities:

Speaking to a section of medical practitioners of Ruvuma Regional Hospital, the Prime Minister ordered that the government will sack practitioners implicated in abortion allegations… He noted that he was informed about practitioners involved in inducing abortion to students and women within the maternal ward, using government equipment/supplies against the public service ethic… (Habari Leo 2016:10, 7 January, authors’ translation).

The above passage voices the government’s official view, i.e. a restrictive stand in line with the country’s abortion law. The newpapers cover several cases where induced abortions have been reported to the police, suggesting that the law in certain cases is actively employed:

(The) Police force in Tarime Rorya had put in custody a nurse from Tarime District Hospital on allegation of inducing abortion to a secondary school girl, 19 years of age. The Police was informed by citizens in the area… The Police took the girl to the hospital for further investigation… The accused practitioner and the girl shall appear in court upon the completion of the investigation (Uhuru 2009:5, 4 December, authors’ translation).

In the interview round a few cases of abortion related prosecution of individuals were brought up. A key informant with long experience from NGO-based sexual and reproductive health programmes said:

Like four or five years ago, a friend of mine who is a doctor was sentenced to 14 years in jail because he was implicated in an abortion case… The girl’s parents became furious and reported it to police. After she (the girl) was severely punished and questioned, she mentioned who assisted her, and the doctor was sent to jail (NGO1).

Although prosecutions were at times referred to in the press, little indicates that the law is actively engaged beyond a few cases where the problem is made public and where it hence is difficult to avoid legal investigation and prosecution. Nonetheless, the fact that the law is not entirely dormant suggests that women who attempt to terminate a pregnancy need to carefully navigate within severe legal restrictions. For the discursive landscape to be presented below the law / Penal Code serves as a continuous backdrop and reference.

Importantly, there are diverse texts that complicate the seemingly clear-cut position of the law. The Constitution of the United Republic of Tanzania of 1977 includes a key provision that articulates fundamental rights to life, human dignity, and equality before the law [34] of relevance in this context. Article 14 of the Constitution provides that every person has the right to life and protection of life by society in accordance with the law. Related to Article 14, the Penal Code details that a child becomes a person capable of being killed when it has completely proceeded in a living state from the body of its mother, regardless of whether it is breathing, has independent circulation or not [33]. This statement appears at stark odds with the abortion law, and is commonly referred to in the contestation surrounding ‘when life begins’ and ‘whose life matters most’, the life of the mother versus the life of the child.

Tanzania has moreover ratified a number of UN conventions and regional treaties that affirm women’s and human rights, including the Convention on the Elimination of All Forms of Discrimination against Women (CEDAW of 1979) [35]; the African Charter on Human and People’s Rights of 1981 [36]; and the Protocol to the African Charter on Human and Peoples’ Rights on the Rights of Women in Africa, popularly known as the ‘Maputo Protocol’ of 2003 [37]. Some of these treaties go to quite some length in spelling out the rights of women, including their reproductive rights. Indeed, by ratifying the Maputo Protocol, the government of Tanzania in principle became obliged to ensure that safe and legal abortion is available and accessible on the following grounds; when the pregnancy is a result of rape, sexual assault, incest and when the pregnancy endangers the life and health of the mother physically and/or mentally [37]. These treaties have a quasi-legal status, indicating quite some degree of contradiction and ambiguity surrounding abortion. We shall return to the battles surrounding such texts below.

### The religious anti-abortion discourse

For Christianity and Islam the crux of the abortion issue is the holy nature of life created by God, and both frame abortion as killing of an unborn child. While Christianity holds that human life begins at conception, Islam conceives life to start 120 days after conception, but we will not enter a discussion of the implications of the distinction in this paper. Christianity (61%) and Islam (35%) make up major religious environments in Tanzania, and the Pew Forum’s survey [38] found that religion is very important in the lives of 93% of the Tanzanian population. The religious and moral discourse against abortion is primarily championed by religious leaders and religious organizations, and for the proponents of the anti-abortion position, the abortion law detailed in the Penal Code as well as in religious texts make up the normative textual references.

Pro-Life Tanzania is arguably the most vocal and active stakeholder advocating the anti-abortion in the country. The organization was established in 1994, and operates under the auspices of the Catholic Church. In a semi-structured interview with the leader of Pro-Life Tanzania (who did not wish to remain anonymous), he detailed how the organization has been carefully monitoring global and national policy debates and programmes on sexual and reproductive health since it was established some 25 years ago. The organization has, in collaboration with religious leaders (and on a few occasions with policy makers such as Members of Parliament), been active in countering initiatives in favor of a more permissive abortion policy in Tanzania.

With technical and financial support from US-based Human Life International, Pro-Life Tanzania develops educational materials and publishes books, booklets and brochures and organizes training seminars. Community radios owned by the Catholic and the Lutheran Churches and a continuous presence in mainstream newspapers make up key aspects of the organizations’ dissemination strategy. The aims are to consolidate negative attitudes against the practice in public discourse and to fight attempts at liberalizing the law. A concrete example of mobilization is, for example, the anti-abortion rallies organized on the day dedicated to right-to-abortion initiatives. The organization did moreover actively engage in the mobilization against the domestication of the Maputo Protocol in 2010 and in the lobbying against The Safe Motherhood Bill in 2012.

The pro-life leader explained the organization’s mobilization approach in connection with the safe motherhood bill

Okay, when we had battles we engaged in programmes, a huge campaign, countrywide, and appealed to our religious leaders no matter what denomination or religion.. We appealed to women and young people in the streets, and then we made extensive use of the media.., we even distributed abortion fear to the members of the parliament and to the president and to the ministers.. Yes, we were lobbying, and of course we made press conferences and press releases (RO1).

Pro-Life Tanzania is hardly alone in defending and voicing the religious anti-abortion discourse in the country. Religious leaders, primarily from Christian denominations, continuously articulate statements condemning abortion to their congregations through their regular religious preaching and teaching as captured in the following newspaper excerpts:

The bishop of the Catholic Church in Shinyanga warned that girls who become pregnant in the area should not abort. Speaking to a congregation of secondary school youth who are members of the Tanzania Youth Catholic Society, he pointed out that aborting constitutes a sin of killing an innocent being (Reporter, Majira, May 5, 2005).

The archbishop of the Catholic Church in Morogoro has condemned the habit of inducing abortion which is widespread in the country, and warned that the practice is against the will of God. He added that the act of inducing abortion is deliberate murder (Reporter, Majira, January 8, 2015).

The religious anti-abortion discourse at times goes quite far, naming the pro-safe abortion lobby ‘death ambassadors’ who are encouraging a ‘culture of death’ versus their own anti-abortion position named a ‘culture of life’.

The powerful religiously grounded anti-abortion discourse operates in line with the restrictive Tanzanian abortion law, and its supporters expressed that they used every opportunity to refer to and defend the law. In the process the discourse positioned itself in stark opposition to the human rights and public health discourses.

### The human rights discourse on abortion

The human rights discourse explicitly draws on global and regional conventional norms locating human rights at the crux of concern, in this context women’s right to health and to decide over their bodies. The discourse is backed by women’s rights activists and human rights organizations, both national and international, a large number being physically present within the NGO environment in Dar es Salaam and in other major cities in Tanzania.

In defense of women’s rights, proponents of the rights based discourse challenge the restrictive, anti-abortion based legal regime encountered in the country, which denies women the right to terminate an unwanted pregnancy. Among the rights based organizations we encountered a discursive shift away from legal restrictions towards legal rights, promoting the liberalization of abortion to achieve development outcomes.

As presented under the Tanzanian law and policy section above, for rights-based approaches the Tanzanian Constitution, national health and development policies and strategies, along with the international declarations, conventions and protocols to which Tanzania is a signatory, constitute the strategic and normative frame of reference. The latter texts in particular, bring women’s rights to the forefront of attention. With reference to such UN and regional human rights instruments, activists on women’s reproductive rights - commonly with the support from their international partners - have during the past three to four decades produced a strong counter narrative which challenges the Tanzanian legal and religious anti-abortion discourse and associated legal practices. An example of a key achievement made by the proponents of the human rights discourse on abortion in Tanzania was the ratification of the Maputo protocol in 2007. As spelled out above, by ratifying the Maputo Protocol, the government of Tanzania in principle became obliged to ensure that safe and legal abortion is available and accessible on a number of grounds.

International institutions advocating for women’s reproductive rights, for example the Centre for Reproductive Rights (CRR) and International Planned Parenthood Federation (IPPF), have provided technical support to local organizations advocating for the provision of safe abortion services. Technical and material is moreover availed under the auspices of United Nations human rights bodies such as the Committee on Economic, Social and Political Rights, and the Special Rapporteurs on the Right to the Highest Attainable Standard of Health and the Rights of Women in Africa. Related support may be encountered in regional bodies such as the African Commission on Human and Peoples’ Rights (ACHPR).

A strong example of how the pro safe abortion discourse manifested itself was the African Commission on Human and People’s Rights (ACHPR) launching of the ‘Decriminalization of abortion in Africa campaign’ in 2016, to bring attention to unsafe abortion as a threat to the rights of women and girls and their sexual and reproductive health. The campaign emphasized that the laws that criminalize abortion expose women and girls to unsafe abortions with gross implications for maternal health in Africa [39]. The 2017 Bill on Reproductive Health Rights proposed by the East African legislature was launched in response to the ACHPR’s campaign. However, although access to abortion services was initially a part of the Bill, it was later removed by the partner states given its sensitivity. An informant summed up the process in the following manner:

As we speak, the East African community is in the process of enacting a Sexual and Reproductive Health and Right Act in which we as partners under the IPPF (International Planned Parenthood Federation) have been participating fully.. We expected that the MPs (Members of Parliament) of the East Africa legislature could pass the Bill, but unfortunately they postponed it (NGO2).

A Bill to enact the Safe Motherhood Act of 2012 had a few years earlier been drafted and tabled to the Parliamentary Committee for Social Services. However, it did not go through to the next legislative processes because of harsh resistance from the religiously based environment. This Bill (Article 5 section 17) provides for the termination of pregnancy, and was formulated very much in line with the Maputo protocol (Article 14c) [37]. In stark contrast to the formulations in the current Tanzanian Penal Code (sections 150 and 151), the Bill of the Safe Motherhood Act of 2012 (section 17) asserts that;

A pregnancy may be terminated if a health care provider, in consultation with the pregnant woman, is of the opinion that the continued pregnancy would pose a risk or injury to the woman’s physical or mental health, there exists a substantial risk that the fetus would suffer from a severe physical or mental abnormality, the pregnancy resulted from rape or incest… [40].

Three organizations, namely Tanzania Women Lawyers Association (TAWLA), Care International, and White Ribbon Alliances took the lead in preparing and drafting the Bill. The initiative was developed in collaboration with a parliamentary caucus on Safe Motherhood. Although the attempt to pass the Bill was not successful, the engagement strongly demonstrated how both the religious anti-abortion discourse and the rights-based pro-safe abortion environment were subjected to forceful counter narratives.

At the same time as the Bill related to the Safe Motherhood Act of 2012 was prepared, TAWLA in Collaboration with the Center for Reproductive Rights in 2012 submitted supplementary information on the United Republic of Tanzania for review by the UN committee on Economic, Social, and Cultural Rights during its 49th session. TAWLA articulated several concerns, including the lack of access to safe abortion services in Tanzania and what they considered to be inconsistencies, ambiguities and contradictions in laws and policies related to reproductive health rights in the country. They appealed to the international community to exert pressure on the Tanzanian state to redress the gaps and inconsistencies.

Another major example of the active abortion engagement by the advocates of reproductive health rights is evident in the not yet finalized Constitutional review process that was particularly intense between 2013 and 2015. During the process, more than 50 women’s rights organizations across the country formed a coalition named ‘Women and the Constitution Coalition’ lobbying for the inclusion of sexual and reproductive rights in the constitution. Although the provision in support of sexual and reproductive health and rights (SRHR) was not included in the first draft, the coalition was successful in lobbying during the production of the second draft. However, the planned referendum on the Constitutional change was postponed in 2015 and has to date not been held. The postponement was celebrated by proponents of the anti-abortion environment, as the constitutional change called for was considered a potential loophole for pro-abortion laws to become legislated in Tanzania. The incomplete Constitutional review remains a key illustration of the discursive confrontations that have taken place between the rights based pro-safe abortion and the religiously based anti-abortion discourses in the country.

### The public health discourse on abortion

The public health discourse on abortion focuses on securing better health outcomes and saving the lives of women vulnerable to health risks associated with unsafe abortion. As such, the public health discourse promotes safe abortion as a means of addressing a pressing public health problems strongly associated with maternal mortality and morbidity. The public health agenda is closely linked to the rights-based discourse, but it is hardly the same. Many of our informants strongly positioned themselves within the public health discourse, but refrained from supporting the rights-based discourse. Proponents of the public health discourse include not only what our informants talked about as a vast majority of medical practitioners, but also parts of the national and international organizations working to promote maternal health. Public health scholars in the field of maternal health also constitute strategic stakeholders in the production of the public health abortion discourse.

Like supporters of the religious and the rights-based discourses, the public health discourse draws heavily on global, regional and national health commitments underlined in policies, strategies and goals. As a signatory to the Millennium Development Goals (MDGs) and later the Sustainable Development Goals (SDGs), Tanzania has signaled its commitment to reducing maternal, newborn and child deaths and improving the quality of MCH care services. The high priority of maternal health initiatives and targets is reflected in several policy documents produced by the Government of Tanzania.

A snapshot of national policies, strategies and guidelines relevant for reproductive, maternal, child and adolescent health in Tanzania, serves to illustrate the point. The Tanzania Vision 2025 articulates health goals to be achieved by 2025 where access to quality reproductive health services for all, and reduced infant and maternal mortality are among the most important health service goals specified. Similar commitments are underlined in at large number of related national policies and strategies. These include, but are not limited to: the National Health Policy of 1990 and 2007; the National Reproductive and Child Health (TCH) Policy guideline of 2015; The National Guideline on Essential Reproductive and Child Health Interventions in Tanzania of 2003; the Health Sector Strategic Plan IV 2016–2020 (HSSP IV); and the National Road Map Strategic Plan to Improve Reproductive, Maternal, Newborn, Child & Adolescent Health in Tanzania (2016–2020).

Supporters of the public health discourse rally behind the demand for better maternal health outcomes. Here, the commitment to saving the lives of women and girls at risk of unsafe abortion drives the efforts to remove barriers to safe, accessible abortion. To justify the agenda, actors within this discursive regime bring to the forefront the harsh implications and the enormous scale of unsafe abortion found not the least among younger women and girls in Tanzania.

Interviews with health practitioners, albeit merely a handful, indicate that despite the conservative legal context, women seeking either abortion or post-abortion care are commonplace both in private and public health facilities. The health workers interviewed were all aware of the legal restrictions against abortion, but nonetheless considered it unethical to deny the benefits of safe, modern abortion methods to what was formulated as ‘victims of unwanted pregnancy’. A midwife expressed it this way:

We are trained to save life and reduce harm.. When a client comes here seeking abortion services, it is very hard to convince her otherwise, so you know for sure that if she doesn’t get the service here she will surely try any other possible option, and we know they often end up with unsafe abortions which lead to serious morbidity and sometimes death. Knowing that she died because you denied her the service… (HP1).

As stated above, the government of Tanzania is committed to providing comprehensive PAC. In his foreword to the PAC Clinical Skills Curriculum published in 2002, the then Chief Medical Officer of the Ministry of health states that [41]:1;

PAC is a strategy for maternal mortality and morbidity. The overall objective is to reduce maternal death… The MoH is committed to scaling up comprehensive PAC so as to reducing abortion-related maternal mortality and morbidity through training of middle level health service providers…

Building on a comprehensive post-abortion care training programme launched in 2000, the Tanzanian Ministry of Health and Social Welfare has since 2007 been expanding the PAC services to lower level facilities in an effort to increase service availability throughout the country. While these services are to assist women in the post abortion phase of spontaneous or induced abortion, it was noted that health providers of abortion services use the window opened through the PAC services as an avenue to enhance the safety of the services. A health care provider phrased it this way:

In a situation where we are confronted by clients who are desperately demanding to terminate unwanted pregnancies, it is easy to recommend medical abortion to such clients and ask them to (later) seek PAC services (HP2).

Another landmark brought up in the interviews was the approval of Misoprostol first for postpartum hemorrhage in 2007 and then for the treatment of incomplete abortion incidences in Tanzania in 2011 [cf 15]. An informant from the ministry responsible for maternal health noted that partners working with maternal health promotion within the ministry have long been advocating for Misoprostol to be made available in health facilities to ease the access to less unsafe abortion procedures (MIN1).

The Harm-Reduction Strategy was another approach mentioned [17]. The strategy entails the implementation of interventions geared towards reducing the health damage caused by abortion in contexts where access to safe abortion services is prohibited and stigmatized. Women and girls seeking abortion services to terminate an ‘unwanted’ pregnancy are within this strategy provided with counselling to accept and go on with the pregnancy, while the ones who insist on terminating the pregnancy are provided with the ‘best option’ in the form of information about Misoprostol and how to use it to secure a safe procedure. Although the harm-reduction strategy has received limited support from the governmental sector, it was said to gain acceptance within the NGOs sector dealing with PAC services (INGO1). The public health discourse hence seems to create a viable pro safe abortion discourse in a context where the rights based discourse according to informants struggled to resonate with ‘African values’.

## Discussion

Within an illegal and highly restrictive abortion context one may envisage the anti-abortion or ‘pro-life’ voice and sentiment to dominate the discursive landscape. The abortion law indeed does make up a foundation that all the interviewees were aware of, and the powerful anti-abortion voice was strongly manifest in public exchange. But as we have demonstrated above, the anti-abortion discourse did not operate alone. Rather, what we encountered was a complex discursive landscape consisting of partly contradictory, partly overlapping abortion discourses. Studies of ‘abortion regimes’ indeed suggest that it is hardly possible to freeze any given regime under a single label, given the highly ambivalent and contested nature of the issue of induced abortion [see 1, 4]. The global rights based discourse arguing for women’s right to abortion, as well as the public health voice calling for reproductive health services - including abortion services - to reduce maternal mortality and morbidity, were encountered alongside the anti-abortion discourse. What already early in the fieldwork struck us as encounters with local manifestations of the contrasting global abortion discourses was only strengthened during the course of the fieldwork.

We argue that rather than viewing the Tanzanian abortion regime as conservative-restrictive, it can more fruitfully be viewed as a hybrid discursive regime constituted of multiple and often competing sub-regimes that intersect, as indicated by Woog and Pembe [14]. This highly ambiguous scenario implies that the population is exposed to diverse abortion related information. It facilitates knowledge not only about the illegality and sinfulness of abortion, but about the right to abortion and about abortion procedures that can save the lives of women with unwanted pregnancy.

The discourses positioned themselves vis a vis one another in an interdiscursive landscape. Fairclough’s (26, 27) concept of *interdiscursivity* points to relations that a particular discourse has to other discourses, and calls for an understanding of the multiplicity of frames of reference of a particular discourse. Shaped by the particularities of the Tanzanian socio-political context, we have seen how the diverse abortion discourses comment upon one another, and engage both in long term ideological battles as well as in political combats played out with intensity at particular points in time. The struggle for legitimacy and hegemony indeed becomes rather dramatic at times, made manifest for example during the contests surrounding the Bill related to the Safe Motherhood Act of 2012; the Constitutional review process between 2013 and 2015 and the battle surrounding the launching of the ‘Decriminalization of abortion in Africa campaign’ in 2016.

In these battles the political combats continuously position themselves vis a vis one another. Interdiscursivity in these struggles for hegemony is dependent upon *recontextualisation,* implying that discursive elements from other discourses are imported and subsequently appropriated and exploited within ones own, as detailed by Faifclough [27]. The use and reuse and movement of text, signs and meaning from an original context to another and new context in this manner continuously shape and reshape the opportunities inherent in the diverse discursive encounters.

In the present research material processes of appropriation of discursive elements were made particularly evident through the deliberate and active use the concepts of ‘health’, ‘life’ and ‘rights’ across the discourses. While the prime location of the ‘rights’ concept is located within the human rights discourse, the ‘health’ concept obviously is the crux of the matter within the public health based debates and ‘life’ is a key metaphor employed by the anti-abortion discourse to hail the life of the unborn child. These concepts were nonetheless pragmatically employed within all three discourses, and filled with different content depending on the particularities of encounters and contexts. Such processes of re-contextualization of key concepts is obviously not unique to the Tanzanian abortion discourse, but the particularities of the content and context of the discursive landscape encountered give the discourses a unique local shape and character.

Discursively, the religiously embedded anti-abortion actors held that they cherish ‘a culture of life’ and accuse the proponents of human rights and public health abortion regimes for advancing a ‘culture of death’. Invoking the human rights framework, proponents of the religious discourse powerfully oppose abortion for denying the unborn child the right to live. The anti-abortion discourse attack the public health discourse for establishing a service that promotes more harm than good, − services that promote death rather than health and life. The psychosocial trauma, morbidity and disabilities that a woman who has had an abortion may suffer in the aftermath of an abortion may in such discourse be cited with reference to scientific studies [42, 43], invoking abortion as a threat to the woman’s (mental) health. The health and rights concepts are in this manner re-contextualized, i.e. appropriated by the anti-abortion discourse and located within new contextual frames. In the process the message that is communicated may be fundamentally transformed.

Seen from the position of public health, the restrictive laws on abortion along with religious anti-abortion values, subject women and girls to maternal health risks associated with unsafe abortion [10, 14, 15]. Thus the public health-, like the anti abortion discourse, draws upon the concepts of life, but in this context ‘life’ is re-contextualized and employed to argue for the ensuring of health and the saving of women’s lives through access to safe abortion services. That is, a saving of lives framed within the maternal health agenda. Interdiscursively, actors within both the public health- and human rights abortion regimes promote safe abortion in defense of the health and lives of girls and women who suffer and die from unsafe abortions.

Within the rights discourse the right to health and life of the woman is the prime focus. The proponents of the human rights based discourses accuse the anti-abortion agenda for instituting a restrictive abortion landscape at the expense of girls’ and women’s right to health and life. We argue that the recontextualization of the concept ‘life’ from the ‘life of the foetus’ to the ‘lives of girls and women’, and the effective blending of the rights, health and life concepts, have availed proponents of the two pro safe abortion discourses opportunities to powerfully negotiate in favour of knowledge about and access to safer abortion services also within a restrictive Tanzanian legal context. The recent National Road Map Strategic Plan to Improve Reproductive, Maternal, Newborn, Child and Adolescent Health in Tanzania (2016–2020) is a case in point. When nationally endorsed reports on reproductive, maternal and adolescent health include statements such as the “*right* to *life* and *health* are basic *human rights*” [44]:29, the policy step to secure women’s health and lives through safe abortion may not seem far.

The presence of multiple and simultaneous discourses manifested through formal texts, media postings, everyday struggles or heightened political combats, facilitate an expansion of the knowledge the public is exposed to. The appropriation of the same value laden concepts of health, life and rights by the three discourses will, we argue, over time facilitate a broader conceptualization of these concepts which will be reflected in the ways the public perceive them. Discourse moreover entails action, it is not mere talk or, as Fairclough phrases it, discourse may be ‘operationalized’ or ‘put into practice’. Hence, a hybrid discursive landscape may open new possibilities. The particular manifestations of interdiscursivity encountered within the field of abortion indeed, in our view, invoke and occasion new possibilities. Post abortion care services and the rapidly expanding misoprostol markets are being exploited by health workers to assist girls with unwanted pregnancies. These are powerful indications not only of expanding knowledge, but of emerging opportunities of access to safer abortion services, as indicated in recent publications [45–47].

### Strengths and limitations

We were in this study not able to systematically explore health workers’ or abortion seeking women- and girls’ views and opinions related to how they (potentially) are influenced by and relate to the abortion law and the diverse and simultaneous abortion debates in the country.

It is likely that operating within the contentious landscape of abortion some informants may portray skewed or biased images of their own or their organization’s views, position etc. Such a bias is a relevant concern in any interview-based research, but the challenge seems particularly pertinent when the study topic is of a sensitive kind. The institutions / organizations themselves chose whom to represent them. Hence, we had no control over what informants were chosen for the interviews. However, our informants were by and large representatives of organizations with formal views on abortion. We reviewed the organizations’ internet sites to gain a sense of how they publicly presented their aims and work, which revealed a substantial degree of conformity between the informants’ views and positions and that on the official web pages.

Although we strongly argue for the centrality of newspapers in Tanzanian public discourse, the present research could have benefitted from a study of abortion debates encountered in other social media, including e.g. Facebook, Twitter, and in radio and television programmes. What is more, although the newspaper search was comprehensive, it is impossible to know to what extent what is published in the newspapers is ‘representative’ of public discourse. In the present policy environment, it is likely that there is an emphasis on the restrictive dimensions of the topic in most newspapers, while the pro-safe abortion/ pro-choice voices- and arguments for safe abortions - to some extent are under-communicated. We carried out our study in Dar es Salaam, the largest hub in the country, where the discursive landscape studied is highly complex. It is likely that we would have encountered a somewhat different discursive scenario in other regions although policy and newspaper content is communicated via a number of highly popular local and national radio stations.

While recognizing a number of potential limitations, we simultaneously argue that our triangulation of legal-, policy-, media-, and interview-based sources generated a large data base on the discursive landscape of abortion in Tanzania. This in turn allowed us to scrutinize in a relatively thorough manner how the various discourses played out, intersected and contradicted and with what potential implications in terms of knowledge and practice.

## Conclusion

The present research found that both anti-abortion as well as pro-safe abortion discourses were vocal and active within the highly restrictive legal abortion context of Tanzania. Multiple global abortion discourses strive for hegemony and legitimacy in framing the abortion question also in a Tanzanian context, but have their local manifestations. The paper has demonstrated how contestations within such a hybrid abortion regime are played out in political disputes where all draw upon the concepts of health, rights and life. We argue that such a complex discursive landscape occasions enhanced knowledge- and access scenarios that may ease avenues to safer abortion services. The findings call for renewed attention to the often complex dynamics at work between national abortion laws and actual access to abortion services.

## Data Availability

The archival media based material can be are available in hard copy from the first author. The interview data is not publicly available to protect the study participants’ anonymity.
